# Efficient intracellular delivery makes cancer cells sensitive to nanoemulsive chemodrugs

**DOI:** 10.18632/oncotarget.17536

**Published:** 2017-04-28

**Authors:** Shan Liu, Dilong Chen, Yuming Yuan, Xue Zhang, Yao Li, Shenglei Yan, Jingqing Zhang

**Affiliations:** ^1^ Chongqing Research Center for Pharmaceutical Engineering, Chongqing Medical University, Chongqing 400016, China; ^2^ Tumor Drug Engineering Research Center, Chongqing Three Gorges Medical College, Chongqing 404120, China

**Keywords:** efficient delivery, cancer cells, nanoemulsive chemodrugs, improved bioavailability, in situ absorption characteristics

## Abstract

Evodiamine has been documented to possess activities in numerous cancer cells. Our preliminary study showed that A549 cells were insensitive to evodiamine. In this paper, A549 cells are sensitive to nanoemulsive evodiamine (EVONE) through an efficient intracellular and systematic delivery. EVONE entered tumor cells by energy-dependent and mainly through clathrin-mediated endocytosis. EVONE exerted a higher cytotoxicity in a dose- and time-dependent manner. The enhanced induction of cell cycle arrest was ascribed to the down-regulation of cyclin B and cyclin dependent kinase 1, while the enhanced induction of apoptosis was due to the activation of caspase −3, −8 and −9 and the decreased B-cell lymphoma 2/ assaciated X protein ratio. Furthermore, the *in vivo* kinetic, bioavailability and *in situ* absorption characteristics of EVONE were much better than those of free evodiamine. The cancer cells insensitive to free chemodrugs became sensitive to nanoemulsive chemodrugs.

## INTRODUCTION

Evodiamine (EVO) is a quinolone alkaloid isolated from the unripe fruit of the Chinese herb *Evodia rutaecarpa* [1, 2]. EVO has been regarded as a very promising anti-tumor remedy in recent years, mainly because of its potent antineoplastic activity against different types of tumor cells with little toxicity to normal human peripheral blood cells. EVO has exerted strong antitumor efficacy in a wide variety of cancer cells originally from humans and mice [3–5] but not in all cancer cells. For example, our preliminary experimental results showed that A549 cells were insensitive to free EVO (no cytotoxicity was observed against A549 cells after 72 h of incubation with 20 μM free EVO in 1‰ DMSO, data not shown). The insensitivity of A549 cells to EVO may be ascribed to an insufficient amount of cellular EVO as follows: the primary antitumor mechanisms of EVO are involved with cell cycle arrest and apoptosis of cancer cells [4], thus, an affluent amount of cellular EVO is a precondition for efficiency; only drugs in the dissolved form can penetrate through the bio-membrane and enter the cells [6]. Due to the very low water solubility of EVO (∼3.39 mg/L) [7], few EVO are expected to pass through the cell membrane and become available in the inner cell. Based on the above analysis, we hypothesize that A549 cells become sensitive due to the improved cellular uptake of EVO by loading EVO into a suitable delivery nanosystem and the improved cellular internalization of EVO can enhance the induction of cell cycle arrest and apoptosis in cancer cells, finally boost the antitumor effects.

Over the past decade, a few nanosystems, such as cell-derived exosomes [8], oxidation-responsive polymersomes [9] and photo- and pH dual-sensitive amphiphilic copolymer PEG43-b-P(AA76-co-NBA35-co-tBA9) micelles [10], have been used to deliver proteins, nucleotides and chemical drugs into the cells to meet different needs, but these systems do not include the delivery of inactive chemicals to insensitive cancer cells. Different from solid state nanoparticles, nanoemulsions are nanosized, thermodynamically stable, transparent or translucent colloidal dispersion systems that are usually formed by two immiscible liquids [11]. Nanoemulsions have been widely used to deliver cosmetic ingredients or anti-inflammatory drugs via the transdermal administration route for a long time. In recent years, a few nanoemulsions have been used for cancer therapy and imaging. For example, the tanshinone IIA nanoemulsions increased the cytotoxicity in human bladder cancer T24 cells [12]; the paclitaxel nanoemulsions overcame multidrug resistance in ovarian carcinoma A2780 cells [13]; and the piplartine nanoemulsions enhanced anti-tumor activity in melanoma tumor bearing mice [14]. Compared to solid nanoparticles, liquid nanoemulsions were able to deliver drugs into different cells more easily, including cancer cells. Nanometric emulsions were used to deliver more solid fluorescent silica nanoparticles into HeLa cells [15]; the cationic nanoemulsions were designed to deliver DNA into an embryonic kidney cell line for further transfection studies [16]; and nanoemulsions were used to deliver the theranostic agent, doxorubicin, into the cell nuclei of A2780 ovarian cancer cells with an external ultrasound trigger [17].

In this study, we designed and prepared nanometric emulsions containing evodiamine (EVONE). We confirmed that the nanoemulsions improved the cellular uptake of EVO, which might be related to the phenomenon that the cancerous A549 cells became sensitive to the nanoemulsive chemodrugs (EVONE), while these cancer cells were insensitive to free chemodrugs (EVO). An investigation of cell cycle arrest, apoptosis and relative protein expression was further performed to elucidate the antitumor mechanisms. Our findings indicated that the improved cellular internalization of EVONE, followed by the enhanced induction of G2/M arrest and apoptosis in human lung cancer A549 cells, could finally make the originally insensitive A549 cells became sensitive. Our study provides a new strategy (loading the chemodrugs into nanoemulsions) to broaden the anticancer spectra of anticancer drugs and ease the pressure for developing new anticancer drugs. Furthermore, as an emerging potential chemodrug, the antitumor effects of free EVO have already been widely investigated, while research regarding its carrier systems has been insufficient. Thus, this study may deepen our understanding of the EVO delivery systems.

## RESULTS

### Principal characteristics of EVONE *in vitro*

**Figure 1:**
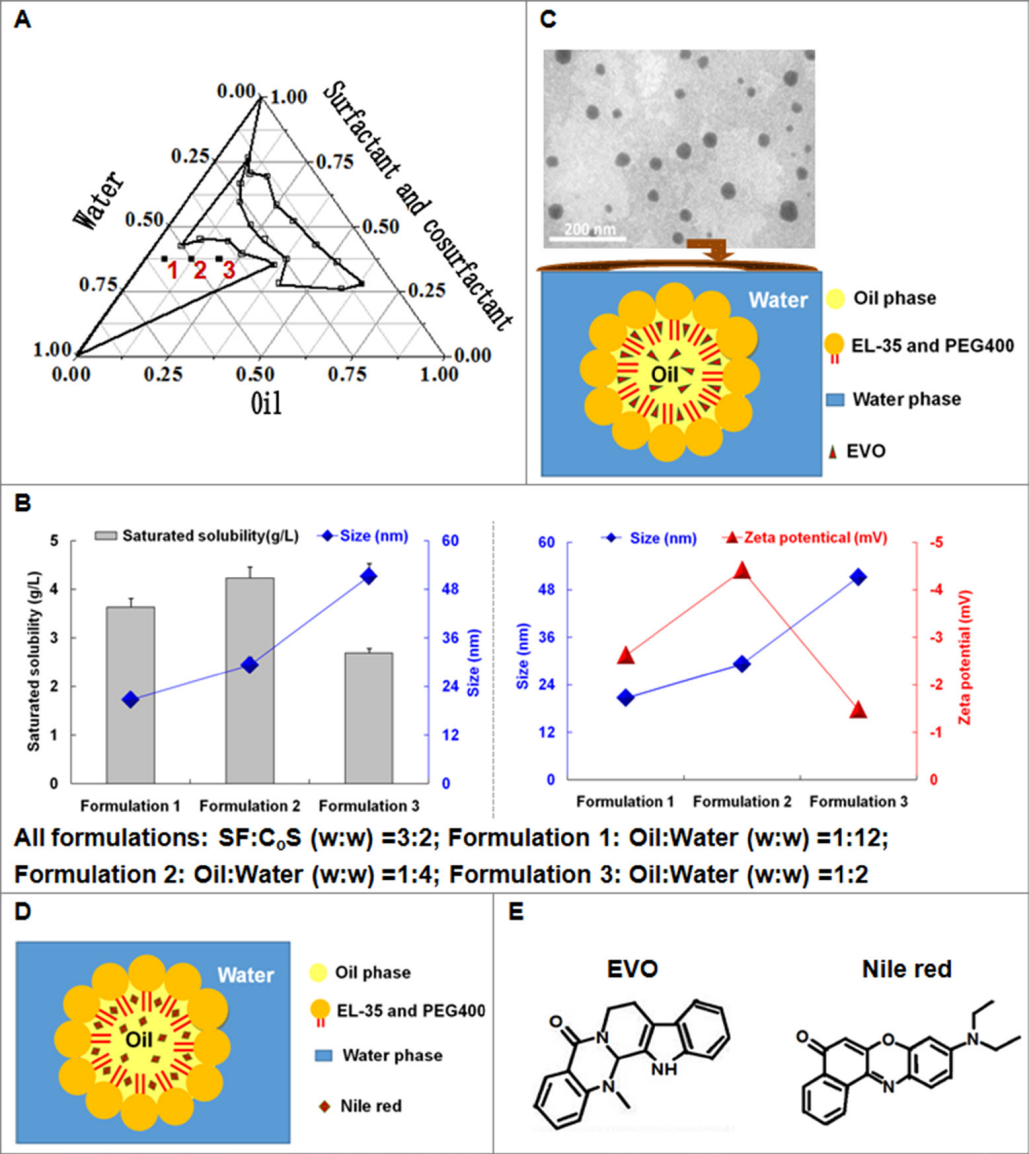
The formulations, physical properties and morphologies of EVONE (**A**) The different compositions of EVONE. (**B**) The effects of compositions of EVONE on the solubility, size, and zeta potential of EVONE. For all formulations: SF:C_o_S (w:w) = 3:2, Formulation 1: Oil:Water (w:w) = 1:11.5; Formulation 2: Oil:Water (w:w) = 1:4; Formulation 3: Oil:Water (w:w) = 1:2. The data were presented as mean ± SD, *n* = 3. (**C**) The transmission election photomicrograph of EVONE and Nile red nanoemulsions. (**D**). prepared using formulation 2. (**E**) Structures of EVO and Nile red. Cremorphor EL 35 and PEG 400 were the surfactant (SF) and cosurfactant (C_o_S), respectively.

EVONE was manufactured using a water titration method. The regions that could form the nanoemulsions are drawn in Figure 1A. Among the representing formulations 1, 2 and 3 (Figure 1B), formulation 2 had the highest solubility and absolute value of zeta potential. EVONE prepared with formulation 2 was prepared for further study. As shown in Figure 1C, all the EVONEs (∼29 nm) were evenly dispersed in the distilled water, and appeared round and intact without aggregation in the TEM image. No visible EVO crystal was present.

### Cytotoxicities of EVONE on the A549 cells

**Figure 2:**
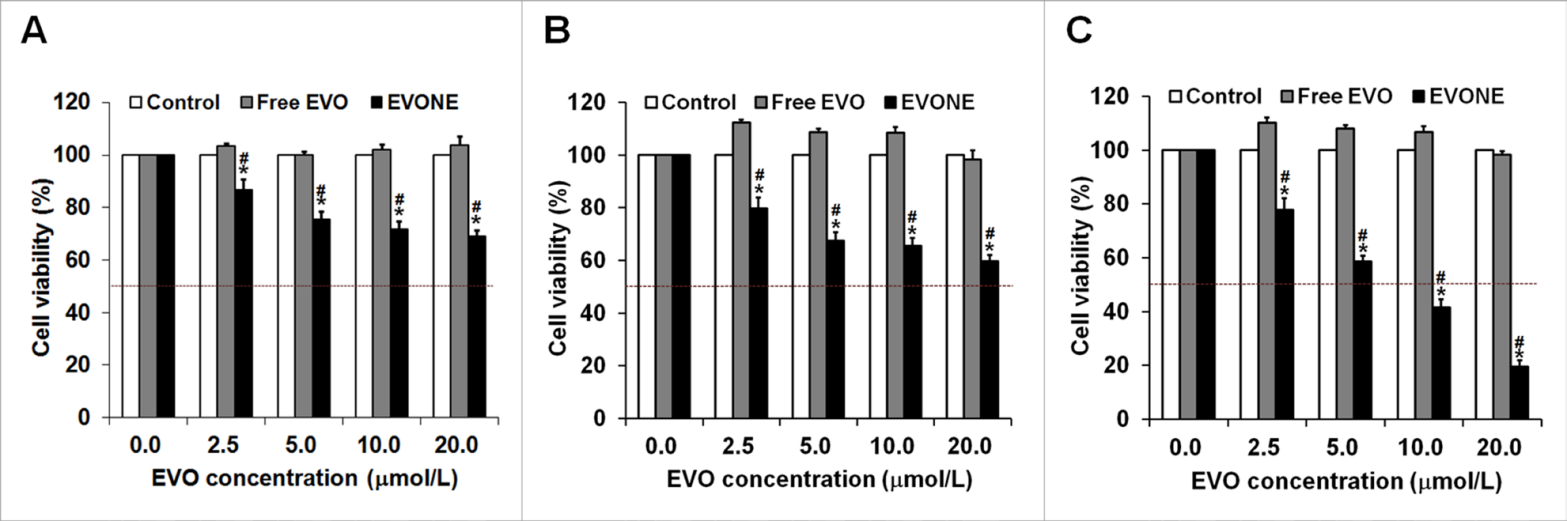
Effects of EVONE on the cell growth of human A549 lung cancerous cells Viability rates of the A549 cells treated with free EVO and EVONE for (**A**) 12 h, (**B**) 24 h and (**C**) 48 h, respectively. Results were presented as the mean ± SD (*n* = 3), *P < 0.05 for the test sample compared with negative control, ^#^*P* < 0.05 for the test sample compared with free EVO.

The cytotoxicities of free EVO and EVONE to A549 cells after treatment for 12 h, 24 h, and 48 h are shown in Figure 2A–2C. The A549 cells treated with free EVO at the concentration of 20 μM for 48 h were insensitive to EVO and remained insensitive even when the incubation time was prolonged to 72 h (data not shown). In contrast EVO incorporated in nanoemulsion (EVONE) exerted cytotoxic effects in the A549 cells in a dose- and time-dependent manner. The cell viability of the A549 cells after treatment with 10 μM EVONE for 48 h was below 50%, and the half-inhibition concentrations (IC_50_) of EVO incorporated in EVONE were 31.53 μM, 17.62 μM and 7.24 μM for 12 h, 24 h and 48 h, respectively.

### Cellular uptake capacity and pathway

**Figure 3:**
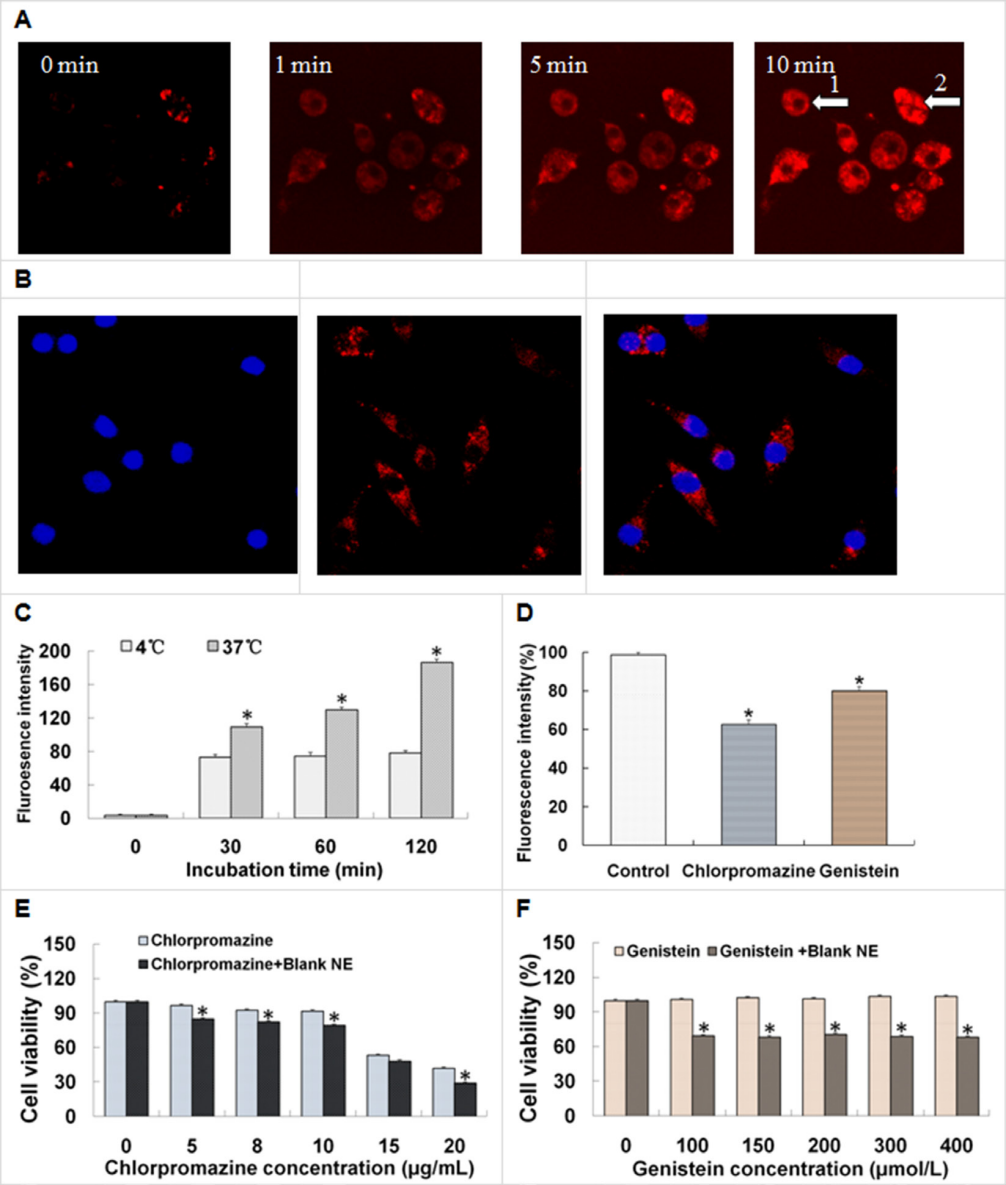
Cellular uptake of nanoemulsion by the A549 cells and the influencing factors (**A**, **B**) Confocal laser scanning photomicrographs of the uptake of nanoemulsions in living A549 cells. Effects of (**C**) temperatures and (**D**) endocytosis inhibitors on the cellular uptake of nanoemulsions. Effects of inhibitors ((**E**) chlorpromazine, (**F**) genistein) and blank nanoemulsion on the viabilities of the A549 cells treated for 3 h, respectively. Results were presented as the mean ± SD (*n* = 3), *P < 0.05 indicated significant differences between the two groups.

A CLSM analysis was performed to qualitatively investigate the cell permeability of nanoemulsion using Nile red fluorescence and Hoechst 33342 blue fluorescence. Figure 3A shows that the amount of cellular Nile red increased quickly within 10 min, reflecting the favorable cell membrane permeability of the nanoemulsion. Figure 3A also indicated that the nanoemulsive systems made it easy to enter the cell nucleus. Figure 3B shows that Nile red fluorescence appeared in all cells but mainly in the cytoplasm. Nile red fluorescence was visible in several cell nuclei (full distribution of red fluorescence in the cells is shown in Figure 3A and the overlap of red and blue fluorescence in the cells is shown in Figure 3B, and the phenomena indicated that nanoemulsion might deliver drugs through some nuclear membranes. The results clearly showed that nanoemulsion was promising for intracellular drug delivery.

As shown in Figure 3C, the cellular uptake of nanoemulsion remained almost unchangeable at 4°C but significantly increased during 120 min (time-dependent) at 37°C. The cellular uptake of nanoemulsion at 37°C was more than twice that at 4°C in 120 min. The significant difference between the cellular uptake at 37°C and 4°C suggested that nanoemulsion entered cells via energy-dependent endocytosis rather than by directly penetrating through the concentration-dependent membrane diffusion. The flow cytometry results showed that the cellular uptake of free Nile red was quite low (data not shown).

Endocytosis inhibition studies were performed to elucidate the cellular trafficking mechanisms of nanoemulsion in A549 cells. Compared with the control, the fluorescence intensities of the cells treated with chlorpromazine and genistein were decreased to different extents (Figure 3D). The chlorpromazine-treated group showed a larger decrease in fluorescence intensities than that of the genistein-treated group (37% versus 20%). Since both chlorpromazine and genistein were inhibitors of the clathrin-mediated endocytosis pathway, the result indicated that the cellular trafficking of nanoemulsions in A549 cells could be mediated by clathrin-mediated endocytosis pathways.

The appropriate concentrations of chlorpromazine and genistein were chosen by evaluating their cytotoxicities in A549 cells. As shown in Figure 3E, chlorpromazine or its mixture with a blank nanoemulsion, possessed remarkable cytotoxicity to A549 cells at certain concentrations (higher than 15 μg/mL), and the presence of Blank nanoemulsion enhanced the cytotoxicity induced by chlorpromazine. Figure 3F shows that genistein was barely cytotoxic to A549 cells, and the mixture of genistein and Blank nanoemulsion somehow exerted a much higher cytotoxicity, but without change, as the genistein concentration increased. The chlorpromazine and genistein concentrations were chosen at 10 μg/mL and 200 μmol/L, respectively, for later use.

### Cell cycle arrest and G2/M cell cycle regulatory proteins

**Figure 4:**
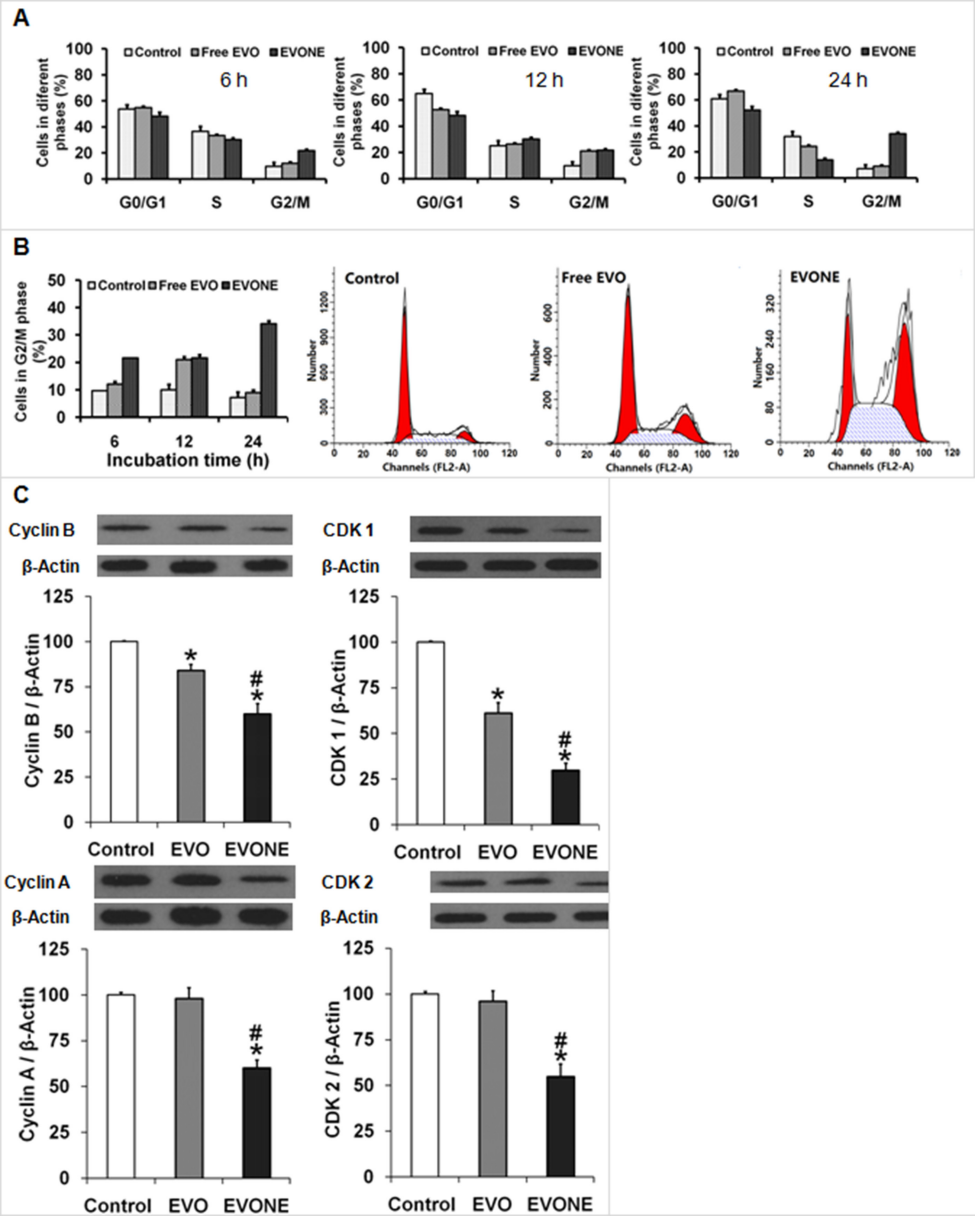
Effects of EVONE on the cell cycle arrest and protein expressions of cyclins and CDCs in human A549 lung cancerous cells Cells arrested in (**A**) different cycle phases or (**B**) G2/M phase of A549 cells when treated with free EVO and EVONE for 6 h, 12 h and 24 h, respectively. (**C**) The protein levels of Cyclin B, CDK 1, Cyclin A, CDK 2. All data were presented as the mean ± SD (*n* = 3), *P < 0.05 indicated significant differences between the test sample and the control, ^#^*P* < 0.05 indicated significant differences between EVONE and free EVO.

The effects of free EVO and EVONE at 10 μmol/L EVO dose on cell cycle progression were examined by flow cytometric analysis. As shown in Figure 4A and 4B, blockage of G2/M phase was observed in the cells treated with EVONE at all three tested time points.At 24 h, the EVONE-treated cells were arrestedin G2/M phase much more than that of free EVO (∼34% versus ∼9%). The expressions of G2/M cell cycle regulatory proteins were then explored. Comparing with the control, the protein levels of Cyclin B and CDK 1 in the A549 cells treated with free EVO or EVONE for 12 h were decreased. EVO might induce G2/M phase arrest via activation of Cyclin B/CDK 1.

### Cell apoptosis and apoptosis-related proteins

**Figure 5:**
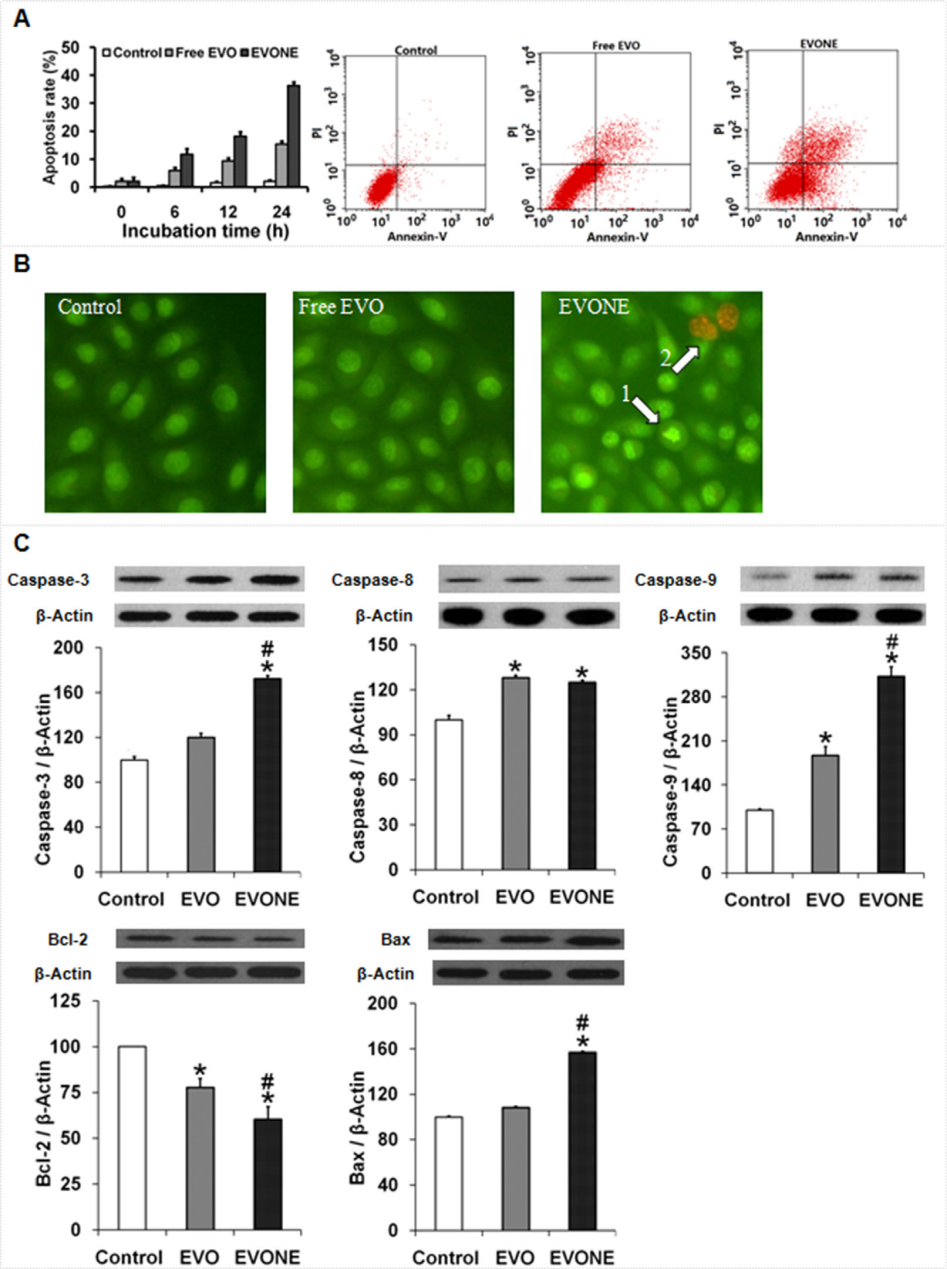
Effects of EVONE on the apoptosis and protein expressions of caspase −3, −8, −9, Bax and Bcl-2 in human A549 lung cancerous cells (**A**) Apoptosis rates of the A549 cells treated with free EVO and EVONE for 6 h, 12 h and 24 h, respectively. (**B**) AO/EB double staining images of the A549 cells treated with free EVO and EVONE. (**C**) The protein levels of caspases −3, −8, −9, Bcl-2 and Bax. All data were presented as the mean ± SD (*n* = 3), *P < 0.05 indicated significant differences between the test sample and the control, ^#^*P* < 0.05 indicated significant differences between EVONE and free EVO.

Both EVONE and free EVO induced cell apoptosis in a time-dependent manner at the concentration of 10 μM (Figure 5A). Compared to the slightly increased apoptosis rates caused by free EVO, the apoptosis rates induced by EVONE were much higher at each time pointwithin 24 h (∼36% versus ∼15%).

The morphologies of A549 cells treated with 2 μM free EVO and EVONE were observed by AO/EB double staining (Figure 5B). After treatment of free EVO for 6 h, A549 cells showed no signs of apoptosis. Incubated with EVONE, A549 cells presented the condensation of chromatin (the condensed green), and formation of apoptosis bodies (the dispersed orange).

As shown in Figure 5C, over-expression of cleaved caspase −3, −8 and −9 in A549 cells treated with EVONE was greatly increased. Modulation of apoptosis regulatory proteins by free EVO was consistent with that of EVONE, but in a much more feeble way. After treatment of EVONE, the anti-apoptotic Bcl-2 protein level was down-regulated, the pro-apoptotic Bax protein level was up-regulated, and the normal expression ratio of Bax/Bcl-2 was accordingly altered. Taken together, members of both caspase family and Bcl-2 family were found to be involved in EVO-induced apoptosis in A549 cells.

### Delivery of EVONE to improve the bioavailability and absorption

**Figure 6:**
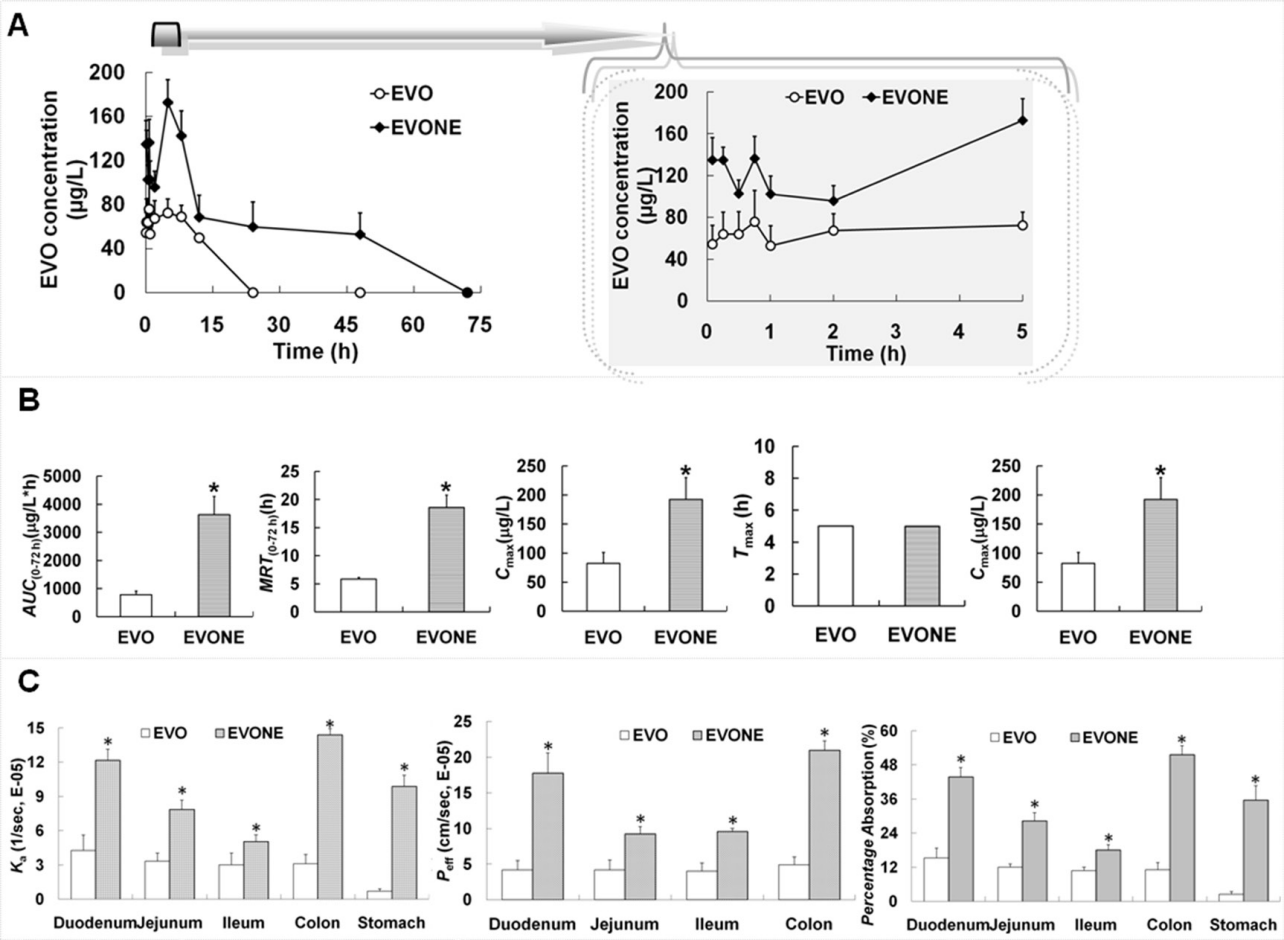
The *in vivo* kinetic and *in situ* absorption characteristics of EVONE (**A**) Plasma evodiamine concentration versus time profiles; (**B**) pharmacokinetic parameters of EVO and EVONE after oral administration; (**C**) absorption rate constant (*K*_a_), effective permeability absorption (*P*_eff_), percentage absorption of EVO and EVONE through gastrointestinal perfusion. The data were shown as mean ± SD. *n* = 6 rats per group. *P < 0.05 indicate significant differences between EVO and EVONE.

Compared to free EVO, EVONE had better kinetic behavior, including higher peak concentration (*C*_max_), longer mean residence time (*MRT*) and lower clearance rate (*Cl*) (Figure 6A and 6B). The relative bioavailability of EVONE to EVO was ∼470%, by comparing their separate *AUC* values.

The absorptive amounts of EVONE in gastrointestinal segments (stomach, duodenum, jejunum, ileum and colon) were markedly increased by 2-14-folds (Figure 6C). The highest increase was found in the colonic section.

## DISCUSSION

During the last decades, drugs discovered from nature have drawn intensive interest [1]. The main alkaloid EVO extracted from traditional Chinese medicine is a good example. The antitumor pharmacology of free EVO has been widely investigated. It was found that EVO had obvious activities on many tumor cells, but not on lung A549 cells in our preliminary study. Thus far, only two delivery systems have been reported to be able to improve the anticancer activities of EVO as follows: one was the EVO/HP-β-CD inclusion complex [18], which exhibited much lower IC_50_ values than free EVO on human hepatoma HepG2 cells for 24 h. The enhanced antitumor effect was ascribed to the increased pro-apoptotic activity and P-glycoprotein inhibition. The other was the poly (lactic-co-glycolic acid) nanoparticles loaded with EVO, which enhanced the proliferation inhibitive effects of EVO on MCF-7 breast cancer cells [19].

However, nanotechnology shows appealing potency in traditional drug design, delivery, medical diagnostics and therapy [20–22]. Among the nanocarriers addressing the obstacles of low-solubility bioactive compounds, nanoemulsions may be superior over micelles, and some other nanocarriers in terms of (1) higher solubilization capacity derived from the inner oil phase besides the surfactant and cosurfactant; (2) easiness and steadiness for sterilization through membrane filtration and autoclave treatment, which is of great importance for experimental and clinical utilization; (3) spherical droplets with tiny size (usually < 100 nm), suggesting huge surface to volume ratios, a very vital property for the efficient delivery of drugs [23]; (4) favorable transparent appearance. Being potential antitumor carriers, nanoemulsions were used to deliver aluminum-phthalocyanine chloride for anticancer photodynamic therapy [24] or deliver perfluorocarbon for ultrasound-mediated tumor imaging and nanotherapy [25]. Most of the time, the nanoemulsions were applied to deliver drugs for direct antitumor therapy without external help as follows: deliver a single chemodrug (e.g., piplartine) [14] or concurrently deliver two chemodrugs (e.g., tocotrienols and simvastatin) [26]; deliver hydrophobic anticancer drugs (e.g., paclitaxel) and/or hydrophilic anticancer drugs (e.g., 5-fluoroucacil) [27]; deliver low molecular chemicals (e.g., paclitaxel) [13], natural compounds (e.g., diallyl disulfide and α-linolenic acid) [28] or DNA plasmid [29] to treat cancer.

Here, nanoemulsions loaded with EVO (e.g., EVONE) were fabricated according to formulation 2. Compared to formulations 1 and 3, formulation 2 had the highest saturated solubility and medium droplet sizes. EVONE was nanosized approximately 29 nm and negative charged about −5 mV. Generally, nanoemulsions loaded with antitumor drugs were very small, e.g., ∼25 nm [24] and ∼80 nm [27]. It was recorded that usually the nanoemulsions loaded with DNA had positive charges (+50 mV) [29], the nanoemulsions loaded with all other kinds of drugs had negative charges (e.g., −6 mV [30] and −12 mV [27]).

Cytotoxicity studies showed that compared with free EVO, EVONE possessed enhanced anti-tumor activities in A549 cells. After 72 h of treatment, 20 μM free EVO completely showed no cytotoxicity to A549 cells, while EVONE showed time- and dose-dependent cytotoxicity to A549 cells with IC_50_ of 17 μM and ∼7 μM for 24 h and 48 h of treatment, respectively. It was documented that nanoemulsions were able to increase the activities of chemodrugs such as tanshinone IIA and dacarbazine [12, 31]. This is the first time that tumor cells became sensitive to nanoemulsions loading with insensentive chemodrugs (i.e., they are insensentive in a free drug state).

We hypothesized that the A549 cells could become sensitive to the insensitive chemo-drug EVO by loading it to a suitable and effective delivery nanosystem (such as nanoemulsion) to achieve markedly enhanced cellular uptake. Moreover, the cytotoxicity of EVONE might be maintained for a long time, once the effective amount of drug was delivered into the cells since EVO would not be transported by efflux P-glycoproteinin some tumor cells such as human multiple-drug resistant breast cancer NCI/ADR-RES cells [32].

The cellular uptake experiments were carried out with CLSM and flow cytometry. Nile red and Hoechst 33342 were selected as the fluorescence probesto stain the cytoplasm [33] or nuclei [34], respectively. Nile red not only had a similar chemical structure to EVO (see Figure 1D and 1E), but also had the ability to dye the intracellular lipids with red fluorescence, while Hoechst 33342 could pass through the hydrophilic nuclear membrane and locate into the cell nucleus. The CLSM images suggested that A549 cells were up-taken with nanoemulsion to a greater extent at a fast rate. Whereas, the fluorescence of cells incubated with free Nile red under the same conditions was too low to be visualized. Reasons for the cellular uptake improvement of EVONE were analyzed as follows: (1) greatly increased solubility (∼4,200 μg /mL of EVONE versus ∼3 μg /mL of free EVO) made it much easier to enter the cell membrane; (2) small droplet size (∼30 nm EVONE versus micro-sized free EVO dispersion) produced more convenient adhesion to, and efficient penetration into the cell membranes; (3) low net value of negative zeta potential with less repulsion between negatively charged cell membrane and droplets (−4.4 mV of EVONE versus −34 mV of free EVO); (4) components in nanoemulsion such as oil or surfactant could enhance the mobility of cell membranes [35].

In addition to the structural and physicochemical characteristics of drug-loaded nanocarriers, the cell type had an influence on the cellular trafficking (or endocytosis) of nanomedicine [36]. In this paper, the A549 cells were selected as the model cells. The reasons are listed as follows: lung cancer was the leading devastating cause of death and there was an urgent need for effective drugs [3]; the non-small cell lung cancer A549 cells derived from human resources were the most common lung cancer model cells recommended by the National Institutes of Health (NIH); EVO exhibited antitumor activities on various cancer cells [3, 5] but not on A549 cells in our preliminary study, while EVO loaded in effective and systemic systems such as nanoemulsions, might offer new strategies to cure lung cancer; the A549 cells were used as *in vitro* models to develop transport protein-aided pulmonary drug delivery systems, such as tacrolimus-loaded nanocomposite microparticles [37].

Compared to the cellular amount of nanoemulsion at 4°C, the amount at 37°C was significantly enhanced, which suggested that the significant difference was induced by temperature. The cellular internalization of nanoemulsion was through energy-dependent pinocytosis in lung A549 cells. Endocytosis was generally classified into phagocytosis (uptake of large particles in specific cell types) and pinocytosis (uptake of fluids and solutes in most cell types), pinocytosis was sub-classified into clathrin-dependent pathway, caveolae-mediated pathway, caveolae and clathrin independent pathway, and macropinocytosis (non-specific pathway). The following two types of chemical endocytosis inhibitors were employed to further elucidate the endocytosis pathways: one was the cationic amphiphilic agent chlorpromazine, which was an inhibitor of the clathrin-mediated endocytosis pathway that translocated membrane-resided clathrin and its adapter proteins to intracellular vesicles to inhibit formation [38]; the other was genistein. It was a tyrosine-kinase inhibitor that inhibited not only the caveolae-mediated endocytosis pathway but also the clathrin- and caveolae-independent endocytosis pathways [39]. Endocytosis inhibition studies showed that cellular trafficking of nanoemulsionswas through multiple endocytosis pathways, predominantly by clathrin-mediated endocytosis. The dimercaptosuccinic acid-coated superparamagnetic iron oxide nanoparticles [40], the mutants of the amphipathic α-helical model peptide (LK peptide) delivering methotrexate and the 1,1,2-triphenyl-2-(p-hydroxyphenyl)-ethene nanoassemblies containing doxorubicin [41] were internalized by clathrin-dependent receptor-mediated endocytosisin breast cancer MCF-7 cells, MDA-MB-231 cells and other cancer cells, respectively.

The anti-tumor activities of EVO were reported to be mediated by the suppression of proliferation and cell cycle arrest, induction of apoptosis, inhibition of invasion and metastasis and blockage of angiogenesis [1]. In this study, it was shown that EVO treatment could down-regulate the expression of cyclin B/Cdk1 complex and induce G2/M arrest in A549 cells, which was inconsistent with a previous report that EVO promoted cell cycle arrest at the G2/M phase in some cancer cell lines [3].

Quantitative cell viability assays and qualitative AO/EB double staining were employed here to compare the anti-tumor activities of EVONE and free EVO. AO/EB double staining is a traditional method for cell apoptosis detection [42]. AO could pass through the membranes of both live and dead cells and emitted green fluorescence, however EB can only penetrate membranes that have lost their integrity and emit orange fluorescence. By AO/EB double staining of A549 cells with 2 μM EVONE for 6 h, normal live cells were stained equally green, early apoptosis cells were stained with condensed green, late apoptosis cells were stained with condensed orange or dispersed orange, and dead cells were stained with equally orange. Here, the condensation of chromatin and the formation of apoptosis bodies occurred, indicating the occurrence of apoptosis; while the morphology of cells treated with free EVO was nearly the same as that of the control. EVO induced apoptosis in A549 cells via altering the protein expression ratio of Bcl-2/Bax and activating caspases −3, −9 and −8. The increased protein levels of caspases −3 and −9 suggested the intrinsic caspase-dependent pathway, while the enhanced caspases-8 level suggested the extrinsic caspase-dependent pathway [3].

Compared with free EVO, the *in vivo* kinetic behavior of EVONE after oral administration has been favorably improved. This study is the first to report a delivery system that is able to simultaneously improve the anticancer activities and pharmacokinetic characteristics of EVO. Only two delivery systems (EVOHP-β-CD complex and EVO poly (lactic-co-glycolic acid) nanoparticles) were documented to improve the antitumor activities of EVO [18, 19], but the *in vivo* kinetic properties of these systems have not been investigated. The markedly enhanced bioavailability of EVONE might be partly ascribed to the increased absorption with a higher absorptive rate and more effective permeability than free EVO. EVO was a substrate of P-glycoprotein in the human hepatoma HepG2 cells [18], while EVONE might protect EVO from deactivation in the gastro-intestinal segments, by entrapping EVO inside the nanoemulsions containing the P-glycoprotein and cytochrome P450 inhibitory surfactants [43]. EVONE also might protect EVO from elimination in systemic circulation before it is released from the nanocarriers.

Further investigations under consideration are listed as follows: in addition to nanoemulsion, other nanosystems, such as lipid nanocapsules, may also be able to make the tumor cells sensitive to chemodrugs since these nanocarriers have some similar characteristics to *in vitro* reactivity and *in vivo* biodistribution [44]; since some nanoemulsion-based formulations such as nanoemulsion-based nanoparticles have more advantages over the drug-nanoemulsion, it is very attractive to further package EVONE in other drug delivery systems to obtain synergistic efficacy [45].

## MATERIALS AND METHODS

### Materials and animals

EVO was provided from Yuancheng Technology Co. Ltd (Wuhan, China). Cremorphor EL 35 was bought from BASF Co. (Ludwigshafen, Germany). Ethyl oleate was purchased from Shanghai Chemical Reagent Co. (Shanghai, China). Polyethylene glycol (PEG) 400 was provided from Tianjinguangfu Fine Chemical Co. (Tianjin, China). The Sprague Dawley rats (200∼250 g) were supplied by the Laboratory Animal Center of Chongqing Medical University. All animal experiments were performed in accordance with the protocol approved by the Laboratory Animal Committee of Medical University.

### Formulation and preparation chosen of EVONE

The nanomeric EVO emulsions were prepared using a water titration method at ambient temperature [46]. Ethyl oleate, cremorphor EL 35 and PEG 400 were selected as the oil phase, surfactant (SF) and cosurfactant (C_o_S), respectively. The nanoemulsive formulation was chosen based on the constructed phase diagram as follows: the weight ratio of SF and C_o_S was fixed at 3:2; the weight ratio of the oil phase to the mixture of SF and CoS was set at 1:9, 2:8, 3:7, 4:6, 5:5, 6:4, 7:3, 8:2, and 9:1. The mixtures of oil, SF and CoS were titrated dropwise with water under gentle magnetic stirring (equilibration should be assured before the next drop). The compositions of the above quaternary mixtures at each clear and cloudy transition point were recorded for the construction of the pseudo-ternary phase diagram (Figure 1A) by Origin software (Version 7.5, Origin Lab Corp., Wellesley, USA). The region enclosed by the zigzag lines (Figure1A) was defined as the nanoemulsion area, and three formulations (see the red solid squares in Figure1A) were selected for further determination.

### Characterization of EVONE

The saturated solubility of EVO in EVONE was determined. The EVONE was prepared, stored to saturation in the dark for 48 h, centrifuged at 12000 rpm for 15 min, and then the EVO content in EVONE was measured by HPLC at 225 nm^7^. The droplet size and zeta-potential of EVNES were determined at 25 °C using photon correlation spectroscopy (Zeta-Sizer Nano-ZS90, Malvern, UK). EVNES was diluted to 1:10 with double-distilled water before measurement. The morphology was observed using TEM H-7500 microscopy (Hitachi, Japan). Routinely, EVNES was placed on formvar-coated copper grids, negatively stained, dried, and the remaining EVNES film on the grids was observed.

### Cytotoxicity study of EVO and EVONE

The effects of free EVO and EVONE on the viability of A549 cells were investigated using the 3-(4,5-Dimethyl-2-thiazolyl)-2,5-diphenyl-2H tetrazolium bromide (MTT) assay. The A549 cells were seeded at a density of 1 × 10^4^ cells per well on a 96-well plate. After an overnight incubation for adherence, the media were replaced with media containing various concentrations of free EVO or EVONE. After 12 h, 24 h or 48 h of incubation, the media were removed and 5 mg/mL MTT solution were added to each well. The cells were further incubated for 4 h, and media were replaced with 150 μL DMSO to solubilize the converted formazan. The absorbance was measured at 570 nm with a Bio-Rad Model 550 microplate photometer (Hercules, CA, USA).

### Cellular uptake efficiency and pathway

The entry character of nanoemulsions into A549 cells was assessed using Nile red as a fluorescence probe^47^. The Nile red nanoemulsions were prepared by the above-mentioned method. A549 cells were seeded in 24-well plates with sterile glass slides overnight for attachment. When the cells were at 80% confluency, the serum-containing media were replaced with serum-free media containing Nile red nanoemulsions. After incubation at 37°C for 15 min, the cells on the glass slides were rinsed with PBS and fixed with 4% formaldehyde for 30 min. After rinsing the slides with PBS for another three times, the cell nuclei were stained with 1 μg/mL Hoechst 33342. The cells were rinsed three times with PBS before observation by confocal laser scanning photomicrography. For the 10 minutes of photochronography, the A549 cells were kept alive at ambient temperature without fixation and nuclei staining.

For determining the effect of temperature on the cell uptake, a Nile red PBS solution (1% DMSO) or nanoemulsions containing 1 μg/mL of Nile red were added to the A549 cells at 85% confluency, and incubated for 120 min at 4°C or 37°C in a 12-well plate without serum. The 4°C samples were pre-chilled for 1 h prior to the addition of nanoemulsions and free EVO and maintained at 4°C during the incubation period. The cells were washed three times with PBS at the corresponding temperature, detached by trypsin, centrifuged for 5 min (1000 rpm), resuspended, and analyzed with a FACSCalibur flow cytometer (Becton Dickinson, Franklin Lakes, NJ, USA). The cellular uptake of the nanoemulsion was calculated as the mean fluorescence intensity.

To investigate the uptake pathways of the nanoemulsion, the A549 cells were pre-treated with 10 μg/mL chlorpromazine or 200 μmol/L genistein for 1 h. Then, 1 μg/mLNile red nanoemulsions were added. After 2 h of additional incubation, the fluorescence intensities of each treated group were detected by flow cytometry.

### Cell cycle arrest and related proteins

A549 cells cultured in 25 cm^2^ flasks were treated with free EVO and EVONES (10 μM) for 6 h, 12 h or 24 h. Cells were then harvested by trypsin, washed twice with ice-cold PBS, centrifugated and fixed with 75% ice-cold ethanol at 4°C overnight. After rising twice with PBS, cells were resuspended in a DNA staining solution containing PI (80 μg/mL), RNase A (100 μg/mL), and 0.1% Triton X-100 for 30 min at room temperature in the dark. Finally, cells were analyzed by FACSCalibur flow cytometer equipped with the CellQuest software (Becton Dickinson, Franklin Lakes, NJ).

The cell cycle proteins cyclin B, cyclin-dependent kinase (CDK)1, cyclin A and CDK2 were determined by Western blotting. The A549 cells cultured in 25 cm^2^ flasks were treated with 10 μM free EVO or EVONE for 12 h, collected and measured. These proteins were detected using primary and secondary antibodies (rabbit anti-cyclin B,-cyclin A, and –CDK2, mouse anti-CDK1, anti-rabbit HRP-IgG and anti-mouse HRP-IgG 3% BSA).

### Cell apoptosis and related proteins

Apoptosis was measured with an apoptosis detection kit. The A549 cells seeded in the 25 cm^2^ flasks were treated with free EVO and EVONE (10 μM) for 6 h, 12 h or 24 h. After the treatment, the cells were collected, centrifuged and resuspended in a staining solution containing PI (50 mg/mL) and Annexin V-FITC (25 mg/mL) for 15 min at ambient temperature in the dark. Cells were then resuspended in the binding buffer and analyzed by a FACSCalibur flow cytometer equipped with CellQuest software (Becton Dickinson, Franklin Lakes, NJ).

The apoptotic proteins caspase −3, −8, −9, Bcl-2 and Bax were investigated by Western blotting. The A549 cells treated with 10 μM free EVO or EVONE for 12 h were collected by scrapers and measured according to a previous report [3]. These proteins were detected using primaryand secondary antibodies (rabbit anti-caspase −3, −9, rabbit anti-Bcl-2, mouse anti-caspase-8, mouse anti-Bax, anti-rabbit HRP-IgG and anti-mouse HRP-IgG 3% BSA).

### *In vivo* kinetics and relative bioavailability

The male rats were orally administered with free EVO or EVONE at the same dose (100 mg/kg EVO). Venous blood samples were obtained at determined times and centrifuged at 3000 rpm for 10 min. The blood samples were treated and measured using the HPLC method [7]. The relative bioavailability of EVONE was calculated by dividing the area under the concentration-time (*AUC*) value of EVONE with that of EVO.

### *In situ* gastrointestinal absorption study

All animal experimental protocols were approved by the Laboratory Animal Committee, Chongqing Medical University. They were performed in accordance with the protocol approved by the Laboratory Animal Committee, Chongqing Medical University. The *in situ* gastric and intestinal absorption were performed as previously described [48]. First, the Sprague Dawley rats were perfused with free EVO or EVONE into the stomach and kept motionless for 2 h, and then, the perfusion was drawn out and subjected to HPLC analysis. Second, the intestinal absorption was investigated by separately perfusing free EVO or EVONE into four enteric fractions at flow rates of 0.2 mL/min for 1 h. The absorption rate constant (*K*_a_), effective permeability absorption (*P*_eff_) and the absorptive extent of EVO and EVONE were calculated and compared.

### Statistical analysis

The results were expressed as the mean ± S.D. of at least three independent experiments. Student’s *t-test* was used to compare the mean of each group with that of the control group, and *P* < 0.05 was considered significantly different. The pharmacokinetic and bioequivalence analyses were conducted using the Drug and Statistics software (Mathematical Pharmacology Professional Committee of China, Shanghai, China)

## CONCLUSIONS

**Figure 7:**
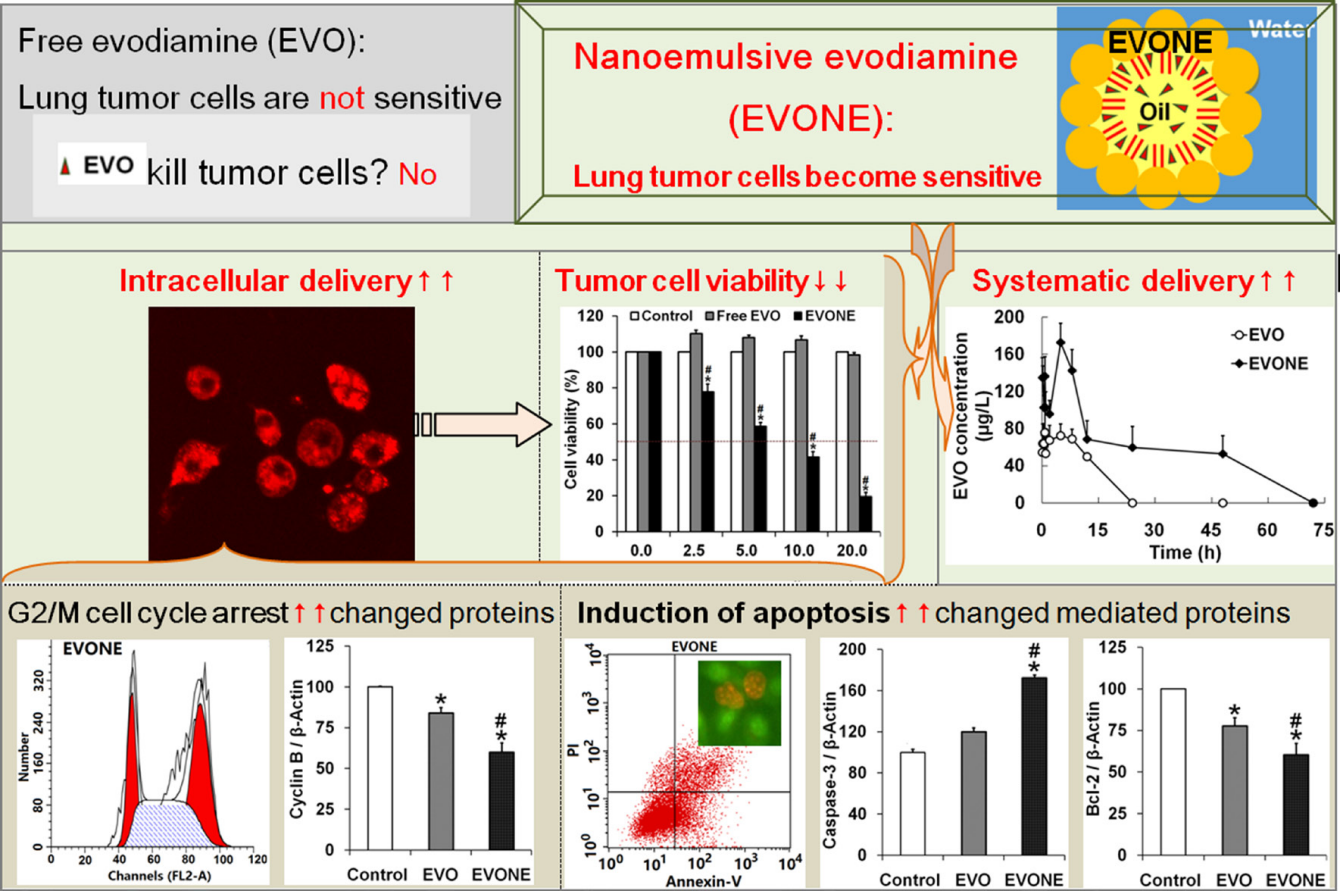
Schematic diagrams of how efficient intracellular delivery make A549 cancer cells sensitive to nanoemulsive EVO with improved bioavailability

EVO was found to be able to produce the previously non-existent anti-tumor activities in human lung cancer A549 cells by being loaded into nanoemulsions (see Figure 7). The emerging anticancer bioactivities of EVONE might be relative to the enhanced cellular uptake (also called cellular internalization), mainly through clathrin-dependent receptor-mediated endocytosis, followed by enhanced induction of G2/M arrest and apoptosis in human lung cancer A549 cells. This study may deepen our understanding of the role of suitable nanosystems in chemo-drug delivery as follows: the cancer cells, insensitive to free drugs, might become sensitive to drug-loaded nanocarriers, such as nanoemulsions. This study reported a novel delivery system able to simultaneously improve the anticancer activities and pharmacokinetic characteristics of EVO, a main constituent from the promising traditional herb Evodia rutaecarpa (Juss.) Benth. This study may bring forward a new perspective for the design of rational delivery systems of EVO.
